# Evaluation of the prehospital administration of tranexamic acid for injured patients: a state-wide observational study with sex and age-disaggregated analysis

**DOI:** 10.1136/emermed-2023-213806

**Published:** 2024-06-14

**Authors:** Camille Girardello, Pierre-Nicolas Carron, Fabrice Dami, Vincent Darioli, Mathieu Pasquier, François-Xavier Ageron

**Affiliations:** 1 Emergency Department, Lausanne University Hospital, Lausanne, Switzerland; 2 University of Lausanne, Lausanne, Switzerland

**Keywords:** trauma, pre-hospital, Observational Study

## Abstract

**Background:**

Tranexamic acid (TXA) decreases mortality in injured patients and should be administered as soon as possible. Despite international guidelines recommending TXA in the prehospital setting, its use remains low. The aim of this study was to assess the prehospital administration of TXA for injured patients in a Swiss region.

**Methods:**

We conducted a retrospective observational study in Switzerland between 2018 and 2021. Inclusion criteria were injured patients ≥18 years for whom an ambulance or helicopter was dispatched. The exclusion criterion was minor injury defined by a National Advisory Committee for Aeronautics score <3. The primary outcome was the proportion of patients treated with TXA according to guidelines. The European guidelines were represented by the risk of death from bleeding (calculated retrospectively using the Bleeding Audit for Trauma and Triage (BATT) score). Factors impacting the likelihood of receiving TXA were assessed by multivariate analysis.

**Results:**

Of 13 944 patients included in the study, 2401 (17.2%) were considered at risk of death from bleeding. Among these, 257 (11%) received prehospital TXA. This represented 38% of those meeting US guidelines. For European guidelines, the treatment rate increased with the risk of death from bleeding: 6% (95% CI 4.4% to 7.0%) for low risk (BATT score 3–4); 13% (95% CI 11.1% to 15.9%) for intermediate risk (BATT score 5–7); and 21% (95% CI 17.6% to 25.6%) for high risk (BATT score ≥8) (p<0.01). Women and the elderly were treated less often than men and younger patients, irrespective of the risk of death from bleeding and the mechanism of injury.

**Conclusion:**

The proportion of injured patients receiving TXA in the prehospital setting of the State of Vaud in Switzerland was low, with even lower rates for women and older patients. The reasons for this undertreatment are probably multifactorial and would require specific studies to clarify and correct them.

**WHAT IS ALREADY KNOWN ON THIS TOPIC**

- Tranexamic acid (TXA) reduces mortality related to post-traumatic haemorrhage but current implementation studies show that the proportion of patients treated is too low.
- Previous implementation studies have assessed the treatment proportions for high-risk injured patients without considering low-to-moderate risk injuries or looking for explanatory factors.

**WHAT THIS STUDY ADDS**

- This retrospective study of the State of Vaud, Switzerland, including a large sample of trauma patients, found that the administration of TXA was low.
- Using the Bleeding Audit for Trauma and Triage score to determine the need for TXA objectively, we found that TXA treatment increased significantly with the risk of bleeding death. However, women and older patients were less likely to be treated, even after adjusting for risk of major bleeding, age and mechanism of injury.
- Although paramedics were required to call a medical team (mobile intensive care unit, MICU) for cases at risk of significant bleeding to administer TXA, MICU dispatch rates were low. Even when MICU was dispatched, treatment levels were suboptimal.

**HOW THIS STUDY MIGHT AFFECT RESEARCH, PRACTICE OR POLICY**

- Further studies are needed to determine why TXA overall is underprescribed and specifically why women and older patients are less likely to receive it.
- Authorising paramedics to give TXA to patients with or at risk of significant bleeding could also help increase the treatment rate.
- As European guidelines do not provide clear guidance, an objective score to guide TXA administration and prevent bias may be useful.

## Introduction

Injuries are one of the leading causes of death worldwide, with more than 450 000 deaths in Europe in 2019.^1^ As haemorrhage is the leading cause of preventable traumatic death,^2^ it is crucial to identify and treat it as soon as possible, starting in the prehospital phase. Tranexamic acid (TXA) has been shown to reduce death in injured patients^3–5^ and should be administered within 3 hours after injury,^6^ as further delay reduces its effectiveness.^5 7 8^ Prehospital administration of TXA is safe^3 9 10^ and allows for a reduction of time to treatment.^11^ However, despite strong evidence of TXA effectiveness in trauma, many injured patients who might benefit from it are not treated.^12–14^

There are multiple reasons why prehospital TXA is underused, including lack of knowledge about TXA, fear of side effects, difficulty in identifying patients at risk of bleeding, lack of specific local protocols and lack of clear treatment criteria.^15^ Different approaches exist between Europe and the USA regarding which patients should be treated. US guidelines recommend a restrictive use of TXA only for high-risk injured patients in the field (systolic BP (SBP) <90 mm Hg and HR>120 bpm).^16^ European guidelines recommend a wider use for injured patients at risk of significant bleeding, that is, as soon as possible, with administration during transport to the hospital.^6^

Previous studies on the implementation of TXA in the trauma population have all involved patients with high-risk injuries, without considering cases with low-to-moderate risk injuries, which represent most of trauma population in EDs. Furthermore, these studies did not assess treatment bias or any explanatory factors for these low treatment rates.^12–14^

The aim of this study was to retrospectively assess the prehospital administration of TXA for a broad cross section of trauma patients and to explore the factors affecting the likelihood of receiving TXA.

## Methods

### Setting

The emergency medical service system of the State of Vaud, Switzerland, serves a population of approximately 800 000 people. It is a two-tier system with a criteria-based dispatch centre staffed by certified nurses and paramedics. Ambulance crews, made up of paramedics, can work autonomously following state protocols to provide intravenous access, administer emergency drugs and initiate and terminate resuscitation. A mobile intensive care unit (MICU) staffed with a prehospital emergency physician may be sent by road or helicopter, by the dispatch centre or at the request of the paramedics on-site. The severity of each case is determined at the end of the mission by the prehospital providers according to the National Advisory Committee for Aeronautics (NACA) score. This score ranges from 0 (no injury or disease) to 7 (death on-site) and describes the patient’s most severe situation during the entire prehospital mission.

Prehospital administration of TXA for injured patients was introduced in European guidelines in 2013,^17^ in US guidelines in 2016^16^ and in the Vaud trauma system in 2015. Indications for the use of TXA in Vaud guidelines are based on the European guidelines^6^ and the inclusion criteria of the clinical randomisation of an antifibrinolytic in significant haemorrhage 2 (CRASH-2) trial^3^ and include injured patients with or at risk of significant bleeding. TXA was initially limited to administration by physicians, but since 2021, use by paramedics is permitted for injured patients with SBP<70 mm Hg if the MICU is unavailable.

### Study design and population

This is a retrospective observational study based on data collected prospectively in the prehospital electronic charts. We included all patients aged 18 years and over who required prehospital care for trauma in the State of Vaud between 1 January 2018 and 30 June 2021. Patients with a NACA score <3 were excluded as they were likely to present minor injuries for which TXA treatment was not indicated.

### Data collection

We collected the following data: sex; age; first prehospital vital signs (SBP, HR, RR, GCS); NACA score; circumstances of the injury; prehospital mortality; prehospital interventions (intubation, vasopressors and TXA administration); transport destination; type of transport (ambulance vs helicopter); prehospital time intervals^18^; and whether a MICU was dispatched or not. TXA administration is a routine variable collected in the patient’s electronic chart. To limit omissions related to TXA administration, we performed keyword searches in the free text in the electronic chart completed by the prehospital providers.

### Outcome and comparison

The primary outcome was the proportion of injured patients receiving prehospital TXA. As not all injured patients benefit from TXA, we analysed the proportion of prehospital treatment by considering the following treatment criteria: US guidelines recommend prehospital TXA treatment for an injured patient with SBP<90 mm Hg and HR>120 bpm,^16^ and European guidelines recommend TXA administration in injured patients with or at risk of significant bleeding.^6^

To represent European guidelines criteria, we assessed prehospital TXA administration according to the baseline risk of significant bleeding using the Bleeding Audit for Trauma and Triage (BATT) score. This score is an internationally validated, prognostic model predicting the baseline risk of death from bleeding with variables available at the injury scene (age, SBP, HR, RR, GCS and mechanism of injury (MOI)).^19 20^ The BATT score was calculated retrospectively for all patients using the data available in the prehospital records (supplemented with imputed data where necessary), and it stratified injured patients into different levels of life-threatening bleeding: unlikely risk (BATT 0–2; risk of death from bleeding <1%); low risk (BATT 3–4; risk of death from bleeding 1%); intermediate risk (BATT 5–7; risk of death from bleeding 5%); and high risk (BATT≥8; risk of death from bleeding 15%). As European guidelines do not specify what constitutes a risk of significant bleeding, we have presented analyses according to three different levels of risk: low, intermediate and high bleeding risk as potential indications for TXA administration. Currently, no prospective studies evaluate the BATT score as a criterion for prehospital administration of TXA, so that the treatment threshold may vary according to the context. In this way, each system can decide the risk level to treat.

Although TXA was not available in every ambulance at the start of the study, all patients had the opportunity to be treated if paramedics identified a significant risk of bleeding and requested a MICU dispatch. This study aims to assess the number of patients treated among the total number of patients for whom treatment was indicated.

### Statistical analysis

We described categorical variables as frequency and percentages and continuous variables as the mean and SD if normally distributed, or as the median and IQR if not normally distributed. We compared categorical variables using Pearson’s χ^2^ test, continuous and normally distributed variables using Student’s t-test and continuous and not normally distributed variables using the Wilcoxon-Mann-Whitney test.

We summarised the characteristics of the study population and assessed TXA administration. We plotted the proportion of prehospital TXA administration by different treatment criteria: (1) US guidelines and (2) European guidelines according to the different levels of the baseline risk of death from bleeding (BATT score) in low, intermediate and high-risk patients.

Exploratory analyses with multivariate logistic regression were performed to identify factors affecting the likelihood of receiving TXA. As potential effect modifiers on TXA administration were expected, interactions between sex, age, MOI and the baseline risk of death from bleeding (based on the BATT score including SBP, HR and GCS) were assessed. We performed a sex and age-disaggregated analysis as recommended by the Sex and Gender Equity in Research guidelines of the European Association of Science Editors.^21^

Missing values for the prehospital vital signs required to calculate the BATT score ranged from 0% to 21%. We performed multiple imputations by chained equations using SBP, HR, RR, GCS, age, the NACA score, early death, intubation and MICU dispatched as covariables. We drew 20 datasets to fill in the missing values. The sample size was fixed due to the retrospective study design. For the explanatory analysis using a multivariate logistic regression model, we were careful not to include many covariables to respect at least 20 events of the outcome per variable. As a sensitivity analysis, we performed a complete case analysis of the main analysis presented in the online supplemental material.

10.1136/emermed-2023-213806.supp1 Supplementary data

## Results

### Description of TXA administration

**Figure 1:**
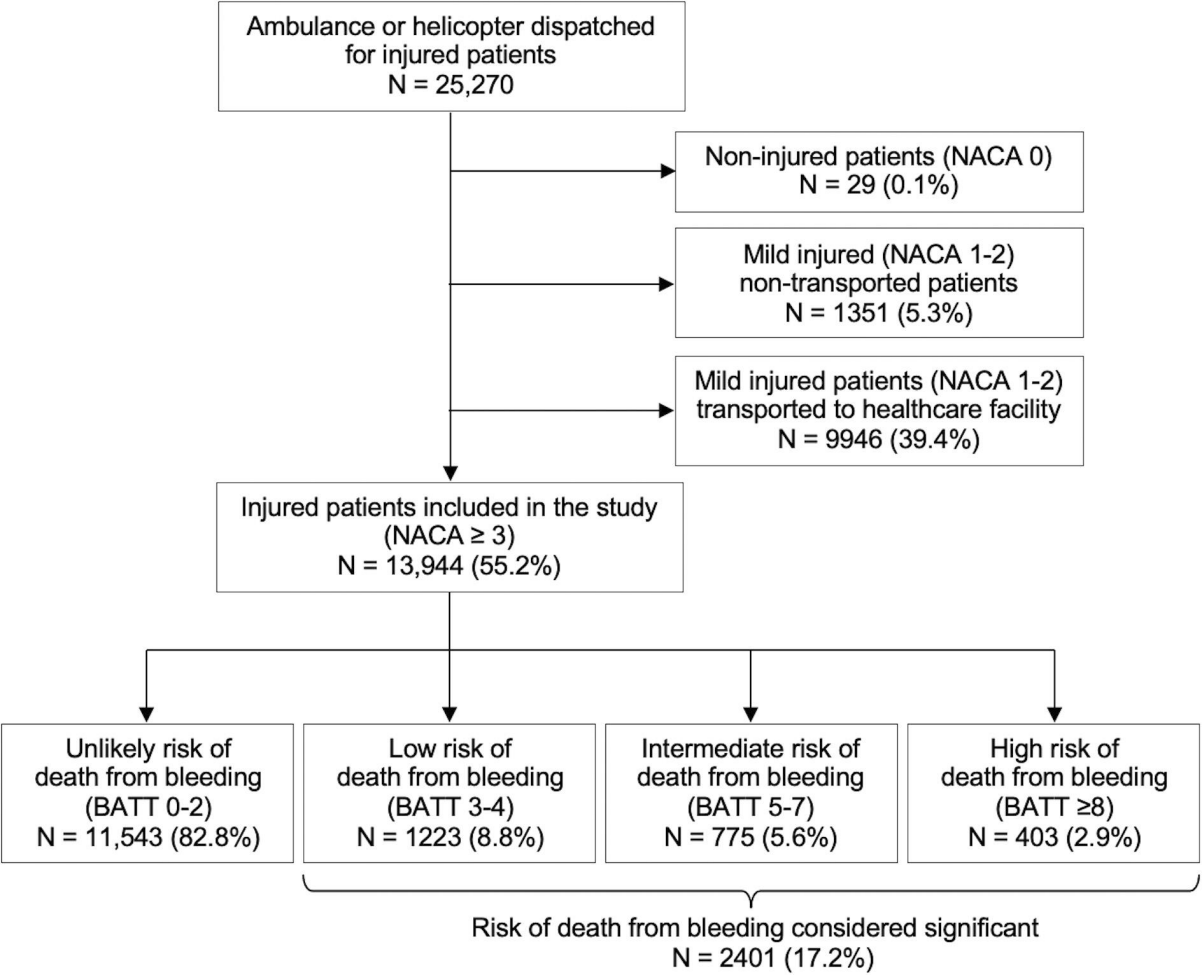
Flow chart of the study population, Vaud, Switzerland, 2018–2021. BATT, Bleeding Audit for Trauma and Triage; NACA, National Advisory Committee for Aeronautics (score)

**Figure 2:**
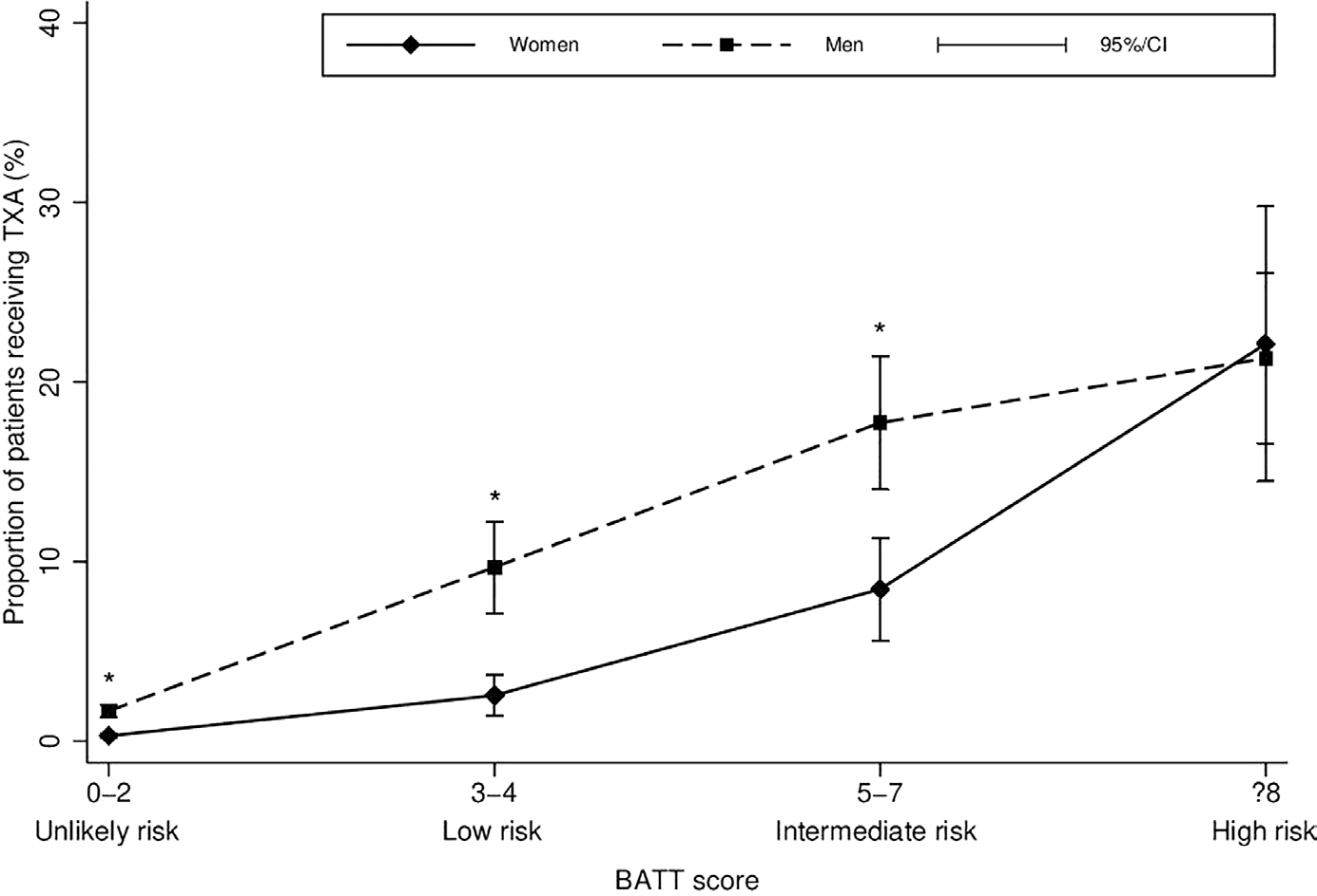
Proportion of tranexamic acid (TXA) administration according to the baseline risk of death from bleeding and sex, Vaud, Switzerland, 2018–2021. *P<0.001. BATT, Bleeding Audit for Trauma and Triage.

**Figure 3:**
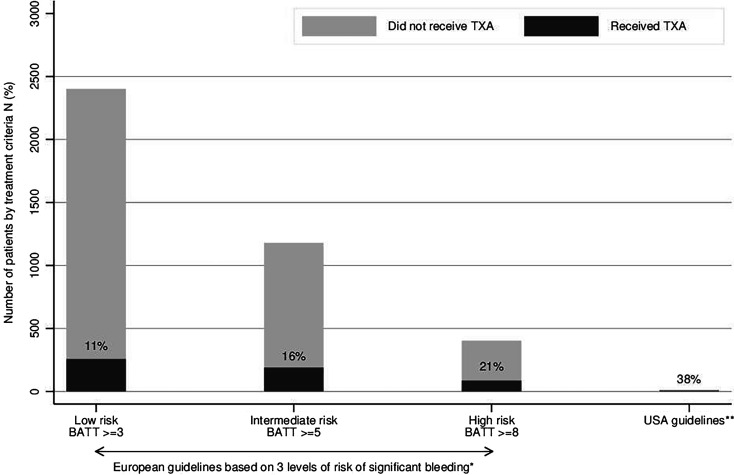
Number and proportion of patients treated by tranexamic acid (TXA) according to different treatment criteria, Vaud, Switzerland, 2018–2021. *Risk of significant bleeding estimated by the BATT score: Bleeding Audit for Trauma and Triage prognostic model. **US guidelines: systolic blood pressure <90 mm Hg and heart rate >120 bpm.

Between 2018 and 2021, an ambulance or helicopter was dispatched for 25 270 injured patients in the State of Vaud. Patients with a NACA score <3 (11 326) were excluded (figure 1). Among 13 944 patients who met the study inclusion criteria, 2401 (17.2%) were considered at significant risk of death from bleeding according to the BATT score (used to represent European guidelines) (figure 1). Among all patients included in the study, 13 (0.09%) met the treatment criteria of the US guidelines, of whom five (0.04%) were treated. Patient characteristics are shown in table 1, and prehospital interventions are shown in table 2. The proportion of patients who received prehospital TXA increased significantly with the increased risk of death from bleeding, ranging from 6% for those at low risk to 21% for high-risk patients (p<0.01) (figure 2, with complete case sensitivity analysis in online supplemental file 1). Table 2 highlights that even in the case of MICU dispatch, treatment rates remain low in all risk categories, varying between 22% and 29% depending on the haemorrhagic risk. The proportion of patients treated with TXA was significantly lower for women than men (74 women (6.2%) vs 183 men (15.1%); p<0.001), except for high-risk (BATT≥8) patients where the treatment rate was similar for both sexes (women 22% vs men 21%; p=0.86) (figure 2). Figure 3 illustrates TXA administration for patients fulfilling different treatment criteria categories. Irrespective of the treatment criteria, the proportion of patients treated was low, and this was also true for patients for whom a MICU was dispatched (online supplemental file 2). Since 2021, only two patients fulfilled the criteria for the paramedic administration of TXA (ie, injured patients with SBP<70 mm Hg), both having a MICU on-site.

**Table 1:** Patient characteristics, Vaud, Switzerland, 2018–2021

	Missing n (%)	Total (n=13 944)	Risk of death from bleeding*		
			Low (n=1223)	Intermediate (n=775)	High (n=403)
**Sex (male), n (%)**	7 (<0.1)	6277 (45)	516 (42)	406 (53)	286 (72)
**Age (years), mean (SD)**	8 (<0.1)	67 (23)	73 (22)	61 (23)	57 (23)
<40, n (%)		2380 (17)	164 (13)	166 (22)	115 (29)
40–54, n (%)		1678 (12)	67 (6)	134 (17)	74 (18)
55–74, n (%)		2836 (20)	147 (12)	200 (26)	82 (21)
≥75, n (%)		7042 (51)	845 (69)	271 (35)	128 (32)
**First vital signs**					
SBP (mm Hg), mean (SD)	1381 (10)	142 (28)	146 (26)	113 (34)	101 (39)
<90 mm Hg (%)		218 (2)	0 (0)	139 (18)	79 (20)
HR (bpm), mean (SD)	656 (5)	82 (19)	103 (19)	84 (21)	98 (28)
>110 bpm, n (%)		736 (5)	356 (29)	71 (9)	50 (12)
**First GCS (points), median (IQR 25–75)**	1147 (8)	15 (15–15)	15 (15–15)	15 (11–15)	3 (3–10)
3–8, n (%)		430 (3)	23 (2)	160 (21)	247 (61)
9–12, n (%)		184 (1)	30 (3)	98 (13)	19 (5)
13–15, n (%)		12 183 (87)	1104 (90)	448 (58)	70 (17)
**NACA (points), mean (SD)**	0 (0)	3.2 (1)	3.2 (1)	4.0 (1)	5.9 (1)
3 (injury requiring hospital admission), n (%)		12 281 (88)	972 (79)	406 (52)	33 (8)
4 (injury that can deteriorate vital signs), n (%)		955 (7)	199 (16)	134 (17)	30 (7)
5 (injury with acute threat to life), n (%)		345 (2)	46 (4)	137 (18)	87 (22)
6 (transported after stabilisation), n (%)		67 (<0.5)	5 (<0.5)	24 (3)	34 (8)
7 (lethal injury, died on-site), n (%)		296 (2)	1 (0)	74 (10)	219 (54)
**Circumstances, n (%)**	1773 (13)				
Transport accident, n (%)		2891 (21)	285 (23)	209 (27)	166 (41)
Low energy		1505 (11)	58 (5)	40 (5)	1 (0)
High energy		889 (6)	208 (17)	150 (19)	162 (40)
Unspecified		497 (4)	19 (2)	19 (2)	3 (1)
Weapons, n (%)		198 (1)	53 (4)	14 (2)	43 (11)
Firearms		50 (<0.5)	8 (1)	6 (1)	27 (7)
Stabbing		148 (1)	45 (4)	8 (1)	16 (4)
Fall, n (%)		8841 (63)	776 (63)	407 (53)	142 (35)
Low energy		8005 (57)	664 (54)	317 (41)	26 (6)
High energy		529 (4)	99 (8)	79 (10)	114 (28)
Unspecified		307 (2)	13 (1)	11 (1)	2 (1)
Struck/crush, n (%)		241 (2)	14 (1)	11 (1)	11 (3)
**Mechanisms with severity criteria**, **n (%)**		1704 (12)	379 (31)	233 (30)	329 (82)
Penetrating injury		250 (2)	70 (6)	22 (3)	53 (13)
High-energy injury		1483 (11)	321 (26)	219 (28)	285 (71)
**All cause of prehospital death**, **n (%)**		379 (3)	10 (1)	97 (13)	267 (66)

*Risk of death from bleeding according to the BATT score: low (BATT 3–4, risk 1%); intermediate (BATT 5–7, risk 5%); high (BATT≥8, risk 15%).

BATT, Bleeding Audit for Trauma and Triage; GCS, Glasgow Coma Scale; HR, heart rate; NACA, National Advisory Committee for Aeronautics (score); SBP, systolic BP; TXA, tranexamic acid.

**Table 2:** Prehospital interventions, Vaud, Switzerland, 2018–2021

	Total (n=13 944)	Risk of death from bleeding*		
		Low (n=1223)	Intermediate (n=775)	High (n=403)
Prehospital interventions, n (%)				
TXA	361 (3)	68 (6)	103 (13)	86 (21)
Intubation	193 (1)	10 (1)	85 (11)	92 (23)
Vasopressor treatment	196 (1)	5 (0)	58 (8)	129 (32)
MICU dispatched on-site	1996 (14)	311 (25)	360 (46)	349 (87)
TXA if MICU dispatched	356 (17)	68 (22)	108 (29)	84 (24)
Transport destination, n (%)				
Trauma centre	5710 (41)	539 (44)	360 (46)	135 (33)
General hospital	7909 (57)	682 (56)	341 (44)	56 (14)
No transport (patient died on-site)	288 (2)	1 (0)	74 (10)	211 (53)
Type of transport, n (%)				
Helicopter	666 (5)	98 (8)	113 (15)	68 (17)
Ambulance	12 953 (93)	1123 (92)	588 (76)	123 (31)
No transport	325 (2)	2 (0)	74 (10)	212 (53)
Prehospital intervals (minutes), median (IQR 25–75)				
Activation interval	4 (3–6)	4 (3–6)	4 (3–5)	3 (3–5)
Response interval	11 (7–16)	11 (7–15)	10 (6–14)	9 (6–13)
On-scene interval	25 (18–34)	27 (20–38)	29 (21–38)	35 (26–49)
Transport interval	13 (9–19)	13 (8–19)	13 (8–19)	12 (8–17)
Total prehospital interval	52 (40–67)	55 (43–68)	55 (42–69)	61 (47–78)

*Risk of death from bleeding according to the BATT score: low (BATT 3–4, risk 1%); intermediate (BATT 5–7, risk 5%); high (BATT≥8, risk 15%).

BATT, Bleeding Audit for Trauma and Triage; MICU, mobile intensive care unit; TXA, tranexamic acid.

### Factors affecting the likelihood of receiving TXA

Women were less likely to be treated than men, with a crude OR of 0.28 (95% CI 0.22 to 0.35; p<0.001) for TXA administration compared with men. After adjustment for the risk of significant bleeding (including SBP, HR, GCS), age and MOI (high energy and/or penetrating), women were still less treated (adjusted OR 0.75; 95% CI 0.58 to 0.99; p=0.042). We found significant interactions between sex and the risk of significant bleeding (p=0.007), between sex and age (p=0.015), between sex and MOI (p=0.009) and between age and MOI (p<0.001) (online supplemental file 3). When combining interactions between sex, age and the risk of significant bleeding, women were treated less often than men in low and intermediate-risk patients, notably in middle-aged (55–75 years; OR 0.60; 95% CI 0.41 to 0.88; p=0.009) and older women (≥75 years; OR 0.41; 95% CI 0.23 to 0.75; p=0.004) (online supplemental file 4). In general, older women were treated less, regardless of the risk of significant bleeding. The interaction between sex and MOI showed that women were also less treated than men for low-energy trauma, irrespective of age (OR 0.10; 95% CI 0.03 to 0.27; p<0.001). When the MOI was considered as high energy, the OR for TXA administration showed no statistically significant difference in women compared with men (OR 0.90; 95% CI 0.65 to 1.24; p=0.511). TXA administration by sex according to the different circumstances of trauma is summarised in online supplemental file 5, while online supplemental files 6 and 7 describe differences in the MOI by sex and age. Overall, the MICU was less frequently dispatched for injured women (crude OR 0.34; 95% CI 0.31 to 0.38; p<0.001; adjusted OR 0.74; 95% CI 0.65 to 0.85; p<0.001). It was also dispatched less frequently for injured women than for men with low-energy trauma, regardless of age (OR 0.63; 95% CI 0.54 to 0.75; p<0.001). For high-energy trauma and/or penetrating injury, the MICU was dispatched less frequently only in older women (≥75 years; OR 0.54: 95% CI 0.30 to 0.98; p=0.041) (online supplemental file 8).

## Discussion

Only a small proportion of injured patients who might benefit were treated with TXA in our prehospital setting, irrespective of treatment criteria. Only 10–21% of patients were treated, depending on the level of significant bleeding considered for European guidelines (low, intermediate or high risk). US guidelines were more restrictive and would have led to the treatment of only 13 (0.5%) of the 2401 patients considered to be at significant risk of death from bleeding in the European guidelines. Women and elderly patients were treated less often, regardless of the risk of significant bleeding or MOI. These inequities were mainly observed in the low and intermediate-risk categories of significant bleeding.

Previous implementation studies showed similar results with a low proportion of TXA administration.^12–14^ Despite high evidence of effectiveness, the implementation of TXA for injured patients remains suboptimal worldwide. Evidence shows that TXA is effective in various injured populations,^22^ with similar benefits for haemodynamically stable and unstable patients.^23^ Recently, Bivens *et al* estimated that early TXA might save more than 3000 deaths per year in the USA if TXA was more widely given in the prehospital setting,^24^ and they advocated implementing a TXA protocol for paramedics in each state.

European guidelines recommend using TXA in injured patients with or at risk of significant bleeding.^6^ As ‘at risk of significant bleeding’ is subject to different interpretations, many low and intermediate-risk patients were not treated. We have presented the results according to different levels of risk of death from bleeding so that everyone can interpret these results according to what they believe to be a significant risk of bleeding. If the prehospital identification of haemodynamically unstable patients is usually obvious, the discrimination of low and intermediate-risk patients is difficult and might lead to undertriage and undertreatment.^25^

In our study, TXA administration was strongly associated with the MOI and its interaction with sex was significant and confounded the interaction with the risk of significant bleeding. In the absence of obvious abnormal vital signs at scene, we hypothesised that the initial assessment was more based on the MOI than objective criteria based on physiological parameters, patient age and frailty. The lack of objective evaluation may have led to inequities disadvantaging women and older patients.^26^ Due to physiological changes and comorbidities, the elderly present a similar risk of death to younger patients for low-energy MOI, but at different thresholds of physiological variables.^27^ These differences in treatment rates have already been illustrated in a sex-disaggregated analysis in the UK, which showed that women are treated less than men, despite a similar treatment effect.^28^

A previous study assessed barriers and facilitators to the prehospital administration of TXA and described barriers such as difficulty in identifying patients at risk of bleeding, lack of specific local protocols and lack of clear treatment criteria.^15^ Therefore, we believe that we need to change the current protocols by including clearer and less subjective treatment criteria to improve practices. Using a trauma score may be helpful to fulfil the gap and tackle the biases observed in this study on TXA administration.^29^ The BATT score was developed for the prehospital risk stratification of death from bleeding and to improve TXA administration. A BATT score ≥3 has recently been shown to be superior in identifying patients at risk of life-threatening bleeding compared with trauma scores predicting the need for massive transfusion with a low sensitivity for the risk of early death.^25^ Using the BATT score with the decision to treat if the score is ≥3 could be an appropriate treatment criterion in our system, leading to the treatment of <10% of injured patients for whom an ambulance is dispatched. This would allow for broader TXA treatment with a preventive goal that would significantly impact on mortality. Monitoring practices in the prehospital setting is essential to observe practice improvement, and trauma registries should include TXA administration and time to administration data. This is also necessary to explore and understand the barriers used by paramedics and physicians leading to sex and age bias in the treatment of trauma and thus to reduce the associated inequities.

### Strengths and limitations

The main strength of our study is the use of a well-designed inception cohort. A broad cross section of trauma patients is assessed, including cases with a low-to-moderate risk of haemorrhagic death, which were not evaluated in previous implementation studies. Inclusion criteria focused on patients with prehospital management and a NACA score ≥3. Selection bias may have occurred in describing the whole trauma population, but as our aim was to observe the administration of TXA, it is unlikely that the inclusion of all trauma patients would have changed the outcome. In addition, an electronic medical chart is created for all ambulances or helicopters dispatched. Paramedics must complete an electronic form for each patient, including physiological variables, MOI, treatment and prehospital time intervals. This requirement limits the selection bias of the study. Given that we are using the first physiological variables, a measurement error might lead to regression dilution bias for the prognostic model. Misclassification of TXA administration could also occur when an administration has been omitted from the record chart. As missing values might lead to misclassification and selection bias, we chose to perform multiple imputations with the assumption that missing values were at random. However, we used multiple imputations only to estimate the baseline risk of death due to bleeding and not for the outcome. To limit omissions related to TXA administration, we searched for possible oversights using keyword searches in the case descriptions by the prehospital providers.

The CRASH-3 trial provided new evidence for the benefit of TXA during our study period,^4^ and thus we could have expected an increase in TXA administration in 2020, but this was not the case.

The use of TXA was limited to the MICU during most of the study period, which might limit the number of patients treated. Patients without MICU dispatch were not excluded from the study population as all patients had the opportunity to be treated if the dispatch centre or paramedics on-site identified or suspected major trauma.

Finally, our study design was not able to explore the reasons why TXA was not administered. Future qualitative studies should be performed to understand barriers and facilitators to TXA implementation.

## Conclusion

Our findings showed that the proportion of injured patients receiving prehospital TXA treatment was low, even for patients at high risk of death from bleeding, and was even lower in women and the elderly. The reasons for this undertreatment are probably multifactorial and would require specific studies to clarify and correct them. We suggest adapting prehospital guidelines with more accurate and objective treatment criteria, including scores such as the BATT score, to increase the rate of adequate treatment and reduce sex and age inequities. Authorising paramedics to give TXA to patients with or at risk of significant bleeding could also help increase the treatment rate.

## Data Availability

Data are available upon reasonable request.
