# Supplementary material for: Evaluation of the prehospital administration of tranexamic acid for injured patients: a state-wide observational study with sex and age-disaggregated analysis
Source: Emerg Med J. 2024 Jun 14;41(8):452–8. doi: 10.1136/emermed-2023-213806 (PMC11287560; doi:10.1136/emermed-2023-213806)

Supplementary material

Supplemental file 1: Complete case sensitivity analysis (i.e. without the multiple imputation) of the proportion of tranexamic acid administration according to the baseline risk of death from bleeding, sex and age, Vaud, Switzerland, 2018-2021

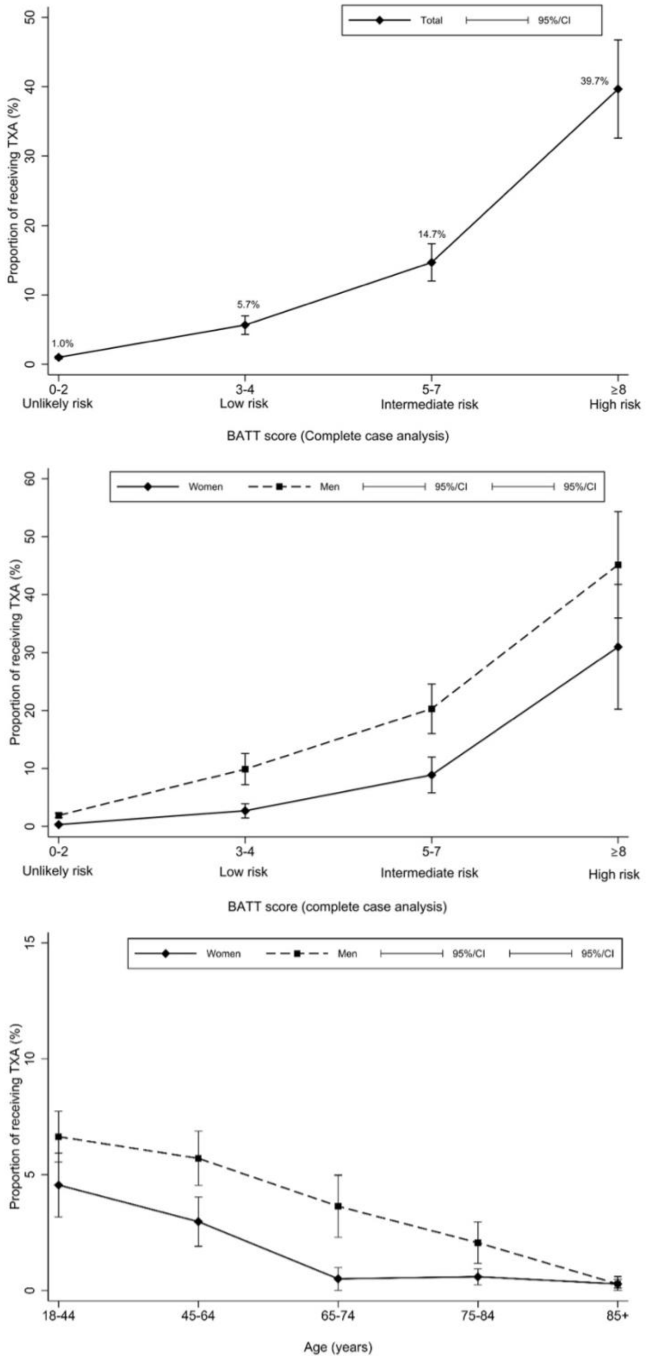

Supplemental file 2: Number and proportion of patient treated by tranexamic acid according to different treatment criteria in the mobile intensive care unit (MICU) group, Vaud, Switzerland, 2018-2021.

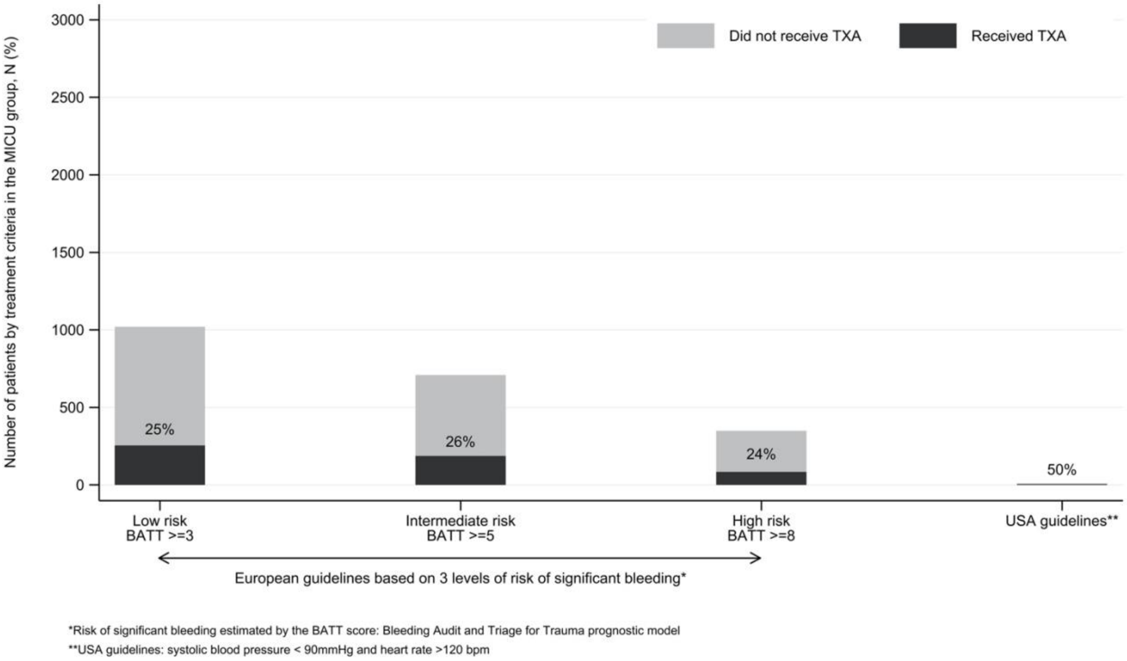

Supplemental file 3: Logistic regression model for tranexamic acid administration, Vaud, Switzerland, 2018-2021

	Odds ratio	95% CI	P value (Wald test)
**Sex, female**	0.75	0.58-0.99	0.042
**BATT score***	2.02	1.54-2.64	<0.001
**BATT score^2 (quadratic)**	0.94	0.90-0.98	0.001
**BATT score^3 (cubic)**	1.00	1.00-1.00	0.006
**High energy trauma**	16.01	11.02-23.38	<0.001
**Penetrating trauma**	7.21	4.39-11.86	<0.001
**Age**	1.05	1.02-1.09	0.001
**Age^2 (quadratic)**	0.99	0.99-0.99	<0.001
**Interaction term**			
**Sex, female x BATT score**	1.14	1.04-1.26	0.007
**Sex, female x age**	1.42	1.07-1.89	0.015
**Sex, female x age^2**	-		0.009
**Sex, female x age^3**	-		0.006
**Sex, female x high energy**	4.81	1.49-15.56	0.009
**Age x high energy**	1.03	1.02-1.05	<0.001

N=13 936, R^2=0.40, auroc=0.95 (0.94-9.96)
*BATT score predicted the risk of death from bleeding and the risk of early death and included systolic blood pressure, respiratory rate, heart rate, Glasgow coma scale.

Supplemental file 4: Gender odds ratio for tranexamic acid treatment by risk of significant bleeding and age, Vaud, Switzerland, 2018-2021

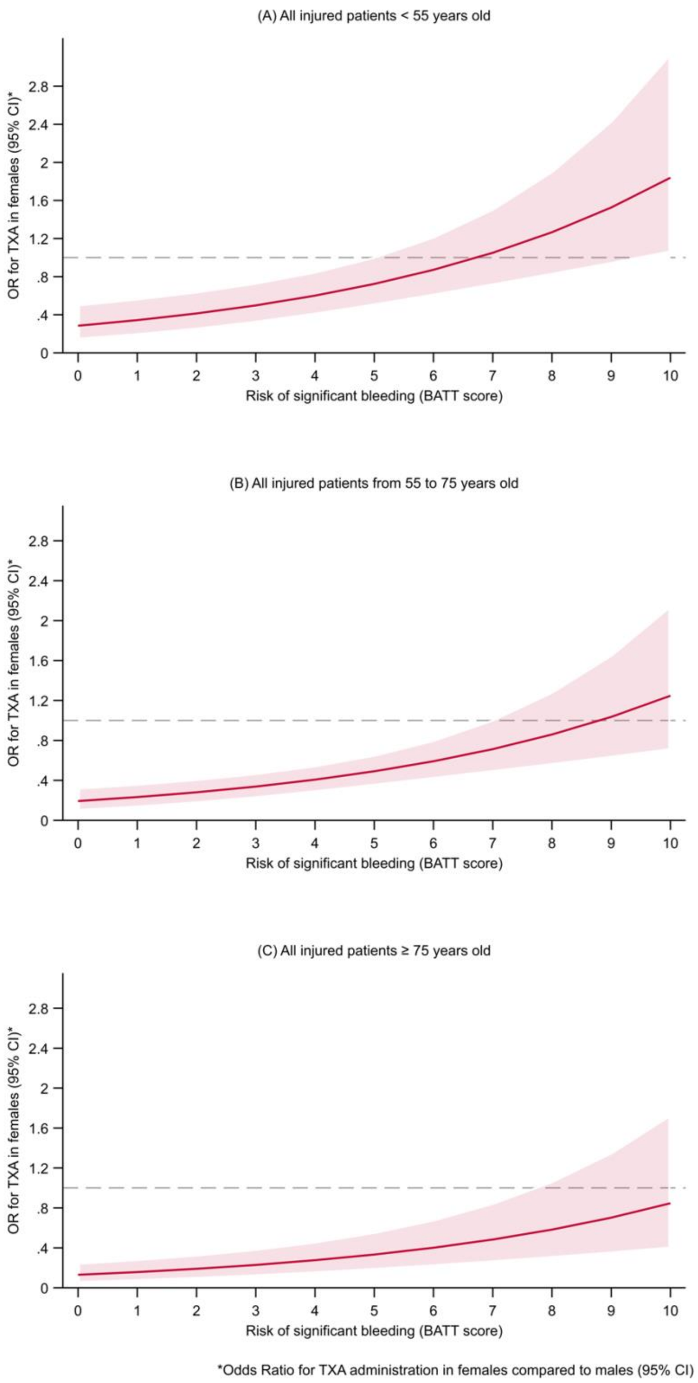

Supplemental file 5: Probability of being treated by mechanisms of injury and sex, Vaud, Switzerland, 2018-2021

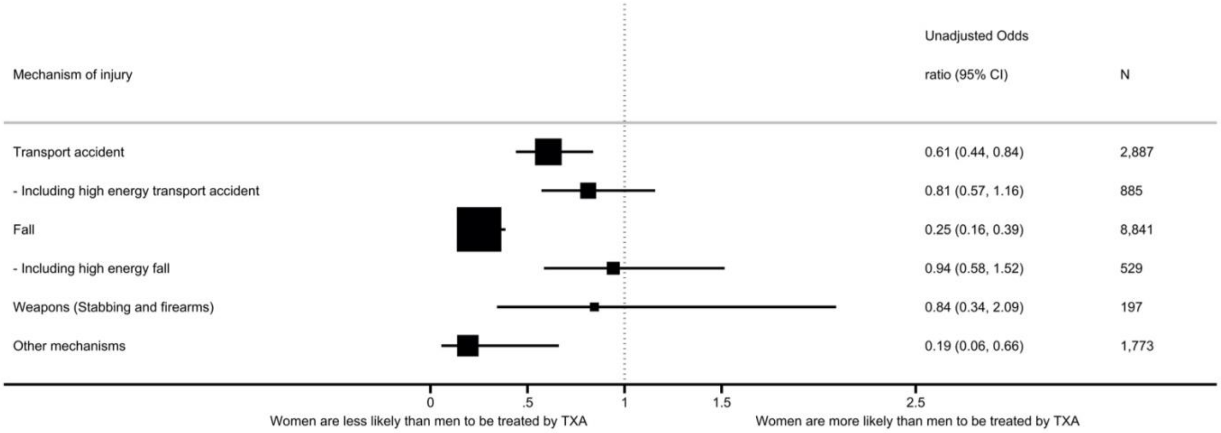

Supplemental file 6: Distribution of injury mechanisms by sex, Vaud, Switzerland, 2018-2021

	**Total (n=13 944)**	**Women (n=7660)**	**Men (n=6277)**
**Transport accident**	2891 (21%)	1027 (13%)	1860 (30%)
**Weapons**	198 (1%)	44 (1%)	153 (2%)
**Struck/crush**	241 (2%)	49 (1%)	191 (3%)
**Fall**	8841 (63%)	5671 (74%)	3170 (51%)
**Unknown**	1773 (13%)	869 (11%)	903 (14%)

Supplemental file 7: Distribution of injury mechanisms by age, Vaud, Switzerland, 2018-2021

	**Total (n=13 944)**	**< 40 years (n=2380)**	**40-54 years (n=1678)**	**55-74 years (n=2836)**	**≥ 75 years (n=7042)**
**Transport accident**	2891 (21%)	1041 (44%)	659 (39%)	697 (25%)	490 (7%)
**Weapons**	198 (1%)	89 (4%)	47 (3%)	31 (1%)	30 (<0.5%)
**Struck / crush**	241 (2%)	149 (6%)	45 (3%)	23 (1%)	23 (<0.5%)
**Fall**	8841 (63%)	666 (28%)	633 (38%)	1713 (60%)	5829 (83%)
**Unknown**	1773 (13%)	435 (18%)	294 (18%)	372 (13%)	670 (10%)

Supplemental file 8: Gender odds ratio for the mobile intensive care unit (MICU) dispatched by age, Vaud, Switzerland, 2018-2021

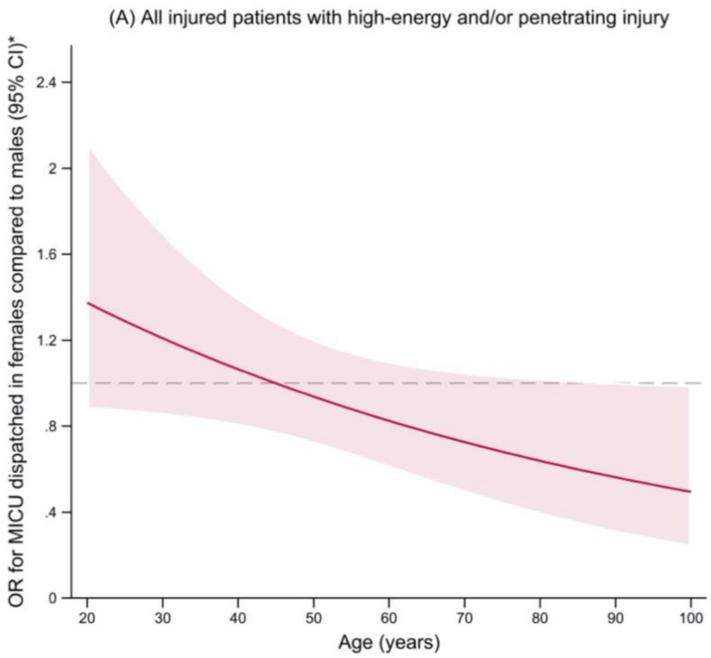

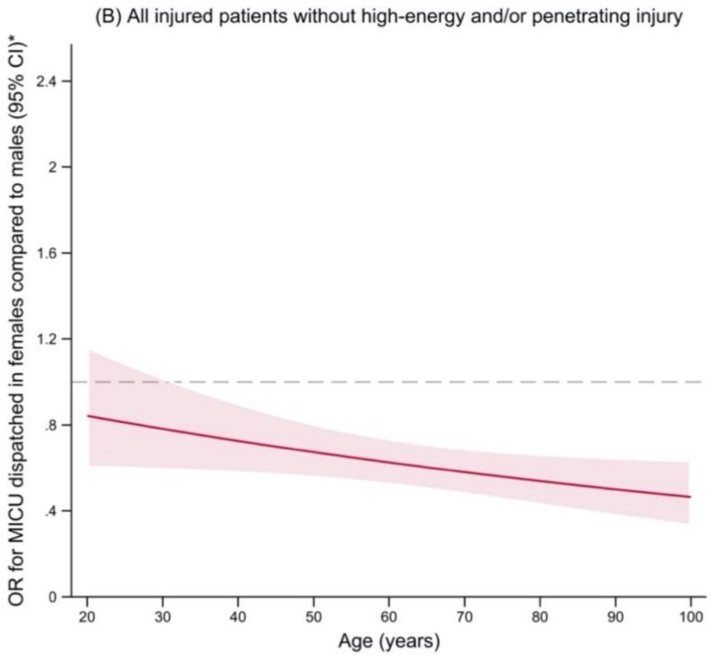

Supplement: Supplementary data [file emermed-2023-213806supp001.pdf]
